# An Association between Maternal Occupations and Low Birth Weight Infants in Japan from 1995 to 2015

**DOI:** 10.3390/ijerph18158040

**Published:** 2021-07-29

**Authors:** Tasuku Okui, Masayuki Ochiai, Naoki Nakashima

**Affiliations:** 1 Medical Information Center, Kyushu University Hospital, Fukuoka 812-8582, Japan; nnaoki@med.kyushu-u.ac.jp; 2 Perinatal and Pediatric Medicine, Graduate School of Medical Sciences, Kyushu University, Fukuoka 812-8582, Japan; ochimasa@pediatr.med.kyushu-u.ac.jp

**Keywords:** vital statistics, Japan, low birth weight, occupations, mothers

## Abstract

Differences in low birth weight rate depending on maternal socioeconomic characteristics have not yet been demonstrated using the Vital Statistics in Japan; therefore, this study aimed to investigate these differences according to maternal occupations. “Report of Vital Statistics: Occupational and Industrial Aspects” and the Vital Statistics in Japan were used every five years from 1995 to 2015. Nine types of occupations were compared. The low birth weight rate was calculated according to maternal occupations and year. Also, the standardized low birth weight ratio was obtained by dividing the number of low-birth-weight infants for each maternal occupation by an expected number of low birth weight infants. The standardized low birth weight ratio for manual workers was the highest among all occupations from 2000 to 2015, and it was significantly higher than one throughout the years. The ratio for clerical workers was also significantly higher than one from 1995 to 2010. Whereas, the ratio for farmers was significantly lower than one in most of the years. It was suggested that health guidance and prenatal care are particularly needed for manual workers, and a study investigating the differences in prenatal characteristics among maternal occupations is necessary for finding a reason for disparity.

## 1. Introduction

Low birth weight is one of the major adverse birth outcomes in perinatal health, and is an important public health indicator [1,2]. Although low birth weight is caused by preterm birth and intrauterine growth restriction, it is known that those infants are at higher risk of perinatal morbidity (such as cardiovascular diseases) and mortality [3,4]. In the world, an estimated low birth weight decreased from 17.5% in 2000 to 14.5% in 2015, and the rate tends to be higher in developing countries [2]. On the other hand, it was shown that the low-birth-weight rate increased from 4.5% in 1979 to 8.3% in 2010 in Japan, and a decrease in energy intake of women and an increase of lean women are considered to be a predisposing factor for this phenomenon [5,6]. Although it is known that the prognosis of low birth weight infants has improved in recent years [7], they are at higher risk of mortality. A study of the characteristics of mothers who give birth to low birth weight infants in Japan is needed to prevent this adverse perinatal outcome.

Low birth weight is known to be associated with the socioeconomic characteristics of women in the world [8,9,10]. In Japan, according to an epidemiological study, high socioeconomic status in the household or employment during early pregnancy increased the risk of low birth weight, and it was found that women tend to be more concerned about their shape when their socioeconomic status is high [11]. Another epidemiological study also revealed that maternal or parental level of education was not significantly associated with very low birth weight [12]. On the other hand, no study investigating the association between low birth weight infants and maternal socioeconomic status using Vital Statistics has been conducted yet in Japan. As Vital Statistics containing socioeconomic factors, “Report of Vital Statistics: Occupational and Industrial Aspects” is often used [13,14]. Maternal occupation is one of the socioeconomic indicators collected in the data. A study investigating the association between fetal mortality rate and maternal occupation has demonstrated that the rates vary with maternal occupations [15], and there is a possibility that maternal occupations affect low birth weight infants too. In addition, a variation in low birth weight rate with maternal occupations has been revealed in Finland [16], whereas no such association has been investigated in Japan. By revealing maternal occupations or socioeconomic statuses of women whose infants tend to be low birth weight in Japan, we could identify target pregnant women for whom health care or guidance is needed and engage in preventive measures.

In this study, we investigated the association between low birth weight and maternal occupations using Vital Statistics by occupation and industry in Japan.

## 2. Materials and Methods

The data of “Report of Vital Statistics: Occupational and Industrial Aspects” in Japan from 1995 to 2015 were used [17]. In Japan, all parents need to submit their infants’ birth certificates to municipalities wherein they live. The birth certificate data were collected by the Ministry of Health, Labor, and Welfare in Japan, and the statistics were published as the Vital Statistics. The Ministry of Health, Labor, and Welfare aims to investigate an association between occupations and demographics, and parents must fill their occupational categories in the birth certificates in years wherein the census are conducted (…, 1995, 2000, 2005, 2010, 2015, 2020, …). Therefore, the Vital Statistics, including the occupational information, are available every five years in Japan, and the data are available in “The Report of Vital Statistics: Occupational and Industrial Aspects”. The percentage of births wherein maternal occupations were unknown was 4.4% in 2005, 4.0% in 2010, and 4.1% in 2015. The number of births wherein maternal occupations were unknown was not published in 1995 and 2000.

Data on the number of births by maternal occupations, maternal age groups, and years and the number of low birth weight infants by maternal occupations and years were publicly available and used in the analysis. Low birth weight infants are infants whose birth weight is less than 2500 g. As the occupation classifications, professional and engineering workers, administrative and managerial workers, clerical workers, sales workers, service workers, security workers, agriculture, forestry and fishery workers, and unemployed persons are available. Regarding manual workers, occupational classifications changed in the analyzed periods, and transport and communication workers, and craft, mining, manufacturing, construction, and labor workers were available in 1995. In 2000 and 2005, a classification of production processes and related workers became available instead of the classification of craft, mining, manufacturing, construction, and labor workers. In 2010 and 2015, these classifications were removed, and manufacturing process workers, transport and machine operation workers, construction and mining workers, and carrying, cleaning, packaging, and related workers, were available. Therefore, we classified these types of workers into one group as manual workers as was done in a previous study [15]. Births whose maternal ages, maternal occupations, or birth weights were unstated or unknown were removed from the data.

In addition, the Vital Statistics data for every five years from 1995 to 2015 were also used [17], and specifically, the data on the number of births and low birth weights by maternal age groups in Japan were used. The number of births by maternal age groups of <20 years, 20–24 years, 25–29 years, 30–34 years, 35–39 years, 40–44 years, and >44 years were available for both the data. Births for which maternal ages or birth weights were unknown were removed from the data.

First, we summarized the number of births and number of low birth weight infants by maternal age group, maternal occupation, and year, using the data of “Report of Vital Statistics: Occupational and Industrial Aspects” and the census data. In addition, low birth weight rate by the maternal age group, maternal occupation, and year were calculated from the result. We also summarized maternal age-specific births by maternal occupation for each year. Then, we calculated the percentage of births for each maternal age group and occupation per year, in order to study the differences in maternal age among occupations.

The low birth weight rate is known to be largely affected by maternal age; as such, it is meaningful to compare the low birth weight rate standardized by maternal age among maternal occupations. However, we could not obtain the data on the number of low birth weight infants by maternal occupations and age groups and could not calculate the low birth weight rate standardized by maternal ages. Therefore, we calculated the standardized low birth weight ratio just like the standardized mortality ratio. To calculate the standardized low birth weight ratio, we calculated the low birth weight rate in all of Japan for each year and maternal age group using the Vital Statistics at first. Then, we calculated an expected number of low birth weight infants for each maternal occupation and year by multiplying the number of births for each maternal age by the maternal age-specific low birth weight rate in all of Japan. The standardized low birth weight ratio was calculated by dividing the number of low birth weight infants for each maternal occupation by the expected number for every maternal occupation and year. All statistical analyses were conducted using R 3.6.3 (https://www.r-project.org/ accessed on 29 July 2021).

## 3. Results

Table 1 shows the number of births, number of low birth weight infants, and low birth weight rate by maternal occupation for each year. The number of births largely varied with the maternal occupation, and the number for unemployed persons was the biggest throughout the years. The number for security workers was the smallest throughout the years. The low birth weight rate has increased from 1995–2015; decreased from 2005–2015 in most of the maternal occupations. The low birth weight rate of manual workers tended to be the highest among maternal occupations.

Table 2 shows percentage of number of births for each maternal age within total number of births for each year and maternal occupation. The percentage tended to be the largest in 25–29 years or 30–34 years, while distribution of the percentages across maternal ages slightly differed depending on maternal occupations. Percentages in older ages tended to be large in administrative and managerial workers.

Table 3 shows the low birth weight rates by maternal age group for each year in all of Japan. The rate tended to be lowest in women aged 25–29 years, and it increased with an increase in maternal age thereafter. In addition, it was found that the low birth weight rate in older age groups decreased from 2005 to 2015.

Table 4 shows the standardized low birth weight ratio and its 95% confidence intervals by maternal occupation for each year. The standardized low birth weight ratio of manual workers was the highest among the different occupations from 2000 to 2015, and it was significantly higher than one throughout the years. The ratio for clerical workers was also higher than one from 1995 to 2010, while it was lower than that of manual workers. On the other hand, the ratio for farmers was significantly lower than one in most of the years, and it was found that the ratio for security workers particularly improved from 1995 to 2015.

## 4. Discussion

We showed differences in low birthweight rates and standardized low birth weight ratios depending on maternal occupations using Vital Statistics data in Japan. As a result, it was found that the standardized low birth weight ratio of manual workers was the highest from 2000 to 2015, and that for agriculture, forestry, and fishery workers remained low. Manual workers were shown to be associated with an elevated risk of low birth weight also in Finland, while farmers and forestry workers also had an elevated risk, which is contrary to the result of this study [16]. We discuss possible reasons for these differences by considering an association between risk factors of low birth weight and maternal occupations in Japan by mainly focusing on manual workers.

Smoking is a risk factor for low birth weight [18], and it was shown that maternal smoking during pregnancy reduced the birth weight of infants in Japan [19]. The smoking prevalence of manual workers is known to be higher than that of workers in other occupations in Japan [20]. The prevalence of passive smoking is known to be high in certain categories of manual workers possibly because they often use a car with multiple employees [21], and the characteristic employment environment of manual workers is considered to be a risk factor for high smoking prevalence [20]. In addition, the educational level is a major factor that explains the association between smoking and occupations. The educational level of manual workers is relatively low compared with the other occupations in Japan [20]. On the other hand, the smoking prevalence of clerical workers, whose standardized low birth weight ratio was relatively high, is the lowest in Japan [20], and factors other than smoking are associated with low birthweight.

Low pre-pregnancy body mass index (BMI) is a risk factor for low birth weight [22]. An increase in the proportion of underweight Japanese women is believed to be a major factor affecting the decrease in the birth weight of infants in Japan [5,6]. According to a previous study on Japanese people, a cluster consisting of mining, transportation, finance, accommodation, and cooperative association was extracted as the high metabolic syndrome group in women [23]. However, there are few studies investigating an association between BMI and occupations in Japan, and there is no significant evidence of an association between underweight and maternal occupations. On the other hand, a positive association between lower educational level and obesity or overweight has been shown in Japan [24].

The use of antenatal care is another factor associated with low birth weight [25,26]. In Japan, the number of times wherein the use of antenatal care is supported by public expenses substantially increased in 2009, and its effect on increasing antenatal care visits and reducing low birth weight rate has been verified in a prefecture in Japan [27]. Although an association between maternal occupation and the utilization of antenatal care has been reported in other countries [28,29], it has not been investigated in Japan. However, the utilization of antenatal care is still not free of charge, and it is considered that low maternal socioeconomic status could lead to the non-use of antenatal care. In addition, the educational level is shown to be associated with antenatal care use in other counties [30], and a reason for this is that women with higher educational levels have more knowledge about health behaviors and have an awareness of the advantage of using antenatal care [30,31,32]. Therefore, there is a possibility that the number of antenatal visits is relatively small in manual workers; therefore, there is a need for a study investigating such an association in Japan. Other than smoking, BMI, and the use of antenatal care, alcohol consumption is reported to be associated with low birth weight or fetal growth restriction in Japan [33,34], and there might exist differences among maternal occupations.

In contrast to manual workers, the standardized low birth weight ratio of agriculture, forestry, and fishery workers remained low throughout the analyzed periods. Although the educational level of agriculture, forestry, and fishery workers is not high in Japan, their smoking prevalence is relatively low among occupations in women [20]. According to a study investigating an association between mean birth weight and household occupations in Japan, the mean birth weight of farmer households was higher than that of other household occupations [35]. As a possible reason for this result, the characteristic family structure of farmer households (living with parents and three generations family) is pointed out [35]. Although it is known that the number of farmer households with three-generation families is decreasing over the years [36], farmer households traditionally tended to be three-generation families. Farmer women might be able to obtain increasing social support by living with their parents or their husbands’ parents. On the other hand, a reason for the amelioration of the standardized low birth weight ratio for security workers is uncertain. Their smoking prevalence does not change much over time [20]. As shown in Table 1, the degree of an increase in the number of births in the periods was relatively high in security workers, and it is considered that the socioeconomic characteristics of mothers who are security workers changed during the study periods. Moreover, unemployed women were not associated with high low birth weight rates, probably because unemployed women were not necessarily poor. It is reported that high income of husbands has a negative impact on the employment status of married women in Japan [37].

It was shown that manual workers had a high standardized low birth weight ratio as compared to the other maternal occupations. The disparity in low birth weight, depending on maternal occupations was revealed. Although it might be difficult to ameliorate the physical differences between mothers depending on maternal occupations, we might be able to change the health behaviors of pregnant manual workers. For example, guidance for smoking cessation is effective not only in prenatal care but also in workplaces. Workplaces are important places for creating awareness that smoking cessation is particularly needed for pregnant women against manual workers. The recommendation for consultation of antenatal care against manual workers in workplaces is also needed. It is considered that establishing a medical staff that workers can consult easily in workplaces is effective for ameliorating these prenatal behaviors in workers. Additionally, antenatal care is not completely free of charge, as previously noted, and making the utilization of antenatal care completely free of charge against low-income households may be effective for easing the disparity among maternal occupations. Moreover, grasping percentage of maternity leave by maternal occupations is also important because maternity leave might be difficult depending on the occupation. If taking maternity leave is difficult for manual workers, we need to create systems for increasing childbearing support in workplaces.

There are some limitations in the current study. First, we could not obtain data on other characteristics of mothers than maternal occupations. As other factors, Vital Statistics collects data on parity, number of pregnancies, legitimacy of child, nationality, gestational ages, birth height, sex, place of residence, and paternal occupation, while individual data containing these factors are not publicly available. Data on these factors can be obtained when using individual data, and an analysis using these factors will be important in the future. In addition, we could not obtain data on the number of low birth weight infants by age group for each maternal occupation, and we could not calculate the low birth weight rate standardized by maternal age. Furthermore, the standardized low birth weight ratio tended to be higher than the average level in clerical workers; however, reason for the phenomenon is uncertain. An accurate reason for the association between maternal occupation and low birth weight is uncertain from this study, and an epidemiological study investigating differences in social and physical characteristics of mothers will be necessary to determine the reason for the disparity.

## 5. Conclusions

We revealed differences in the standardized low birthweight ratio between maternal occupations using Vital Statistics. As a result, the standardized low birth weight ratio of manual workers was the highest in most of the years, and it was significantly higher than one throughout the years. On the other hand, the standardized low birth weight ratio of agriculture, forestry, and fishery workers remained low throughout the analyzed periods. It was suggested that more attention and medical care aimed at preventing low birth weight is needed, particularly in women who are manual workers, and a further study investigating reasons for the association between mothers being manual workers and low birth weight is necessary.

## Figures and Tables

**Table 1:** Number of births, number of low-birth-weight infants, and low birth weight rate by maternal occupations for each year.

	Year				
Maternal Occupation	1995	2000	2005	2010	2015
Number of births					
Professional and engineering workers	80,126	88,852	87,951	117,353	151,190
Administrative and managerial workers	3615	3191	3272	4915	5137
Clerical workers	85,366	85,824	81,634	103,655	129,251
Sales workers	21,230	19,799	18,668	25,328	32,870
Service workers	20,176	22,010	29,602	45,058	65,114
Security workers	1392	1716	2216	2487	2941
Agriculture, forestry, and fishery workers	6650	4386	3589	3904	4193
Manual workers	24,411	18,714	14,937	17,983	21,444
Unemployed persons	922,163	914,112	765,807	693,754	536,683
Number of low birth weight infants					
Professional and engineering workers	6091	8054	8766	11,555	14,220
Administrative and managerial workers	257	277	361	490	502
Clerical workers	6720	7746	8277	10,445	12,598
Sales workers	1540	1755	1741	2402	3061
Service workers	1663	1994	2843	4386	6328
Security workers	109	136	195	201	231
Agriculture, forestry, and fishery workers	450	336	306	348	346
Manual workers	1932	1730	1597	1889	2163
Unemployed persons	69,398	78,597	72,190	66,237	50,071
Low birth weight rate					
Professional and engineering workers	7.60	9.06	9.97	9.85	9.41
Administrative and managerial workers	7.11	8.68	11.03	9.97	9.77
Clerical workers	7.87	9.03	10.14	10.08	9.75
Sales workers	7.25	8.86	9.33	9.48	9.31
Service workers	8.24	9.06	9.60	9.73	9.72
Security workers	7.83	7.93	8.80	8.08	7.85
Agriculture, forestry, and fishery workers	6.77	7.66	8.53	8.91	8.25
Manual workers	7.91	9.24	10.69	10.50	10.09
Unemployed persons	7.53	8.60	9.43	9.55	9.33

**Table 2:** Percentage of number of births for each maternal age within total number of births for each year and maternal occupation.

	Maternal Age Groups						
Year and Maternal Occupation	<20 Years	20–24 Years	25–29 Years	30–34 Years	35–39 Years	40–44 Years	>44 Years
1995							
Professional and engineering workers	0.1	7.6	38.9	39.3	12.7	1.4	0.0
Administrative and managerial workers	0.0	4.6	29.3	41.7	20.7	3.5	0.1
Clerical workers	0.3	12.9	42.8	33.6	9.3	1.1	0.0
Sales workers	0.5	14.7	38.8	32.8	11.2	2.0	0.1
Service workers	1.2	17.1	38.6	30.4	10.6	2.0	0.1
Security workers	0.6	29.8	42.6	22.2	4.3	0.5	0.0
Agriculture, forestry, and fishery workers	0.4	11.7	36.8	35.5	13.6	2.0	0.1
Manual workers	0.8	18.1	43.4	29.1	7.6	1.0	0.0
Unemployed persons	1.6	17.4	41.5	30.6	7.9	0.9	0.0
2000							
Professional and engineering workers	0.1	8.1	36.6	38.2	15.1	1.9	0.0
Administrative and managerial workers	0.0	3.6	23.7	40.9	26.8	5.0	0.0
Clerical workers	0.2	8.0	41.7	36.4	12.2	1.4	0.0
Sales workers	0.6	12.0	37.3	34.0	13.8	2.2	0.1
Service workers	0.9	15.3	37.2	32.2	12.4	1.9	0.1
Security workers	0.9	15.2	51.9	24.1	7.2	0.8	0.0
Agriculture, forestry, and fishery workers	0.5	10.6	34.7	35.9	15.0	2.9	0.2
Manual workers	1.1	15.4	41.1	31.0	10.1	1.3	0.0
Unemployed persons	2.1	14.6	39.5	32.6	10.0	1.1	0.0
2005							
Professional and engineering workers	0.1	5.4	32.3	41.9	17.7	2.6	0.1
Administrative and managerial workers	0.1	2.5	17.4	41.4	30.4	7.6	0.6
Clerical workers	0.2	5.4	28.2	46.1	17.7	2.3	0.1
Sales workers	0.4	10.0	30.9	38.4	17.2	2.9	0.1
Service workers	0.7	12.7	35.1	35.4	13.8	2.4	0.1
Security workers	1.2	12.6	34.9	38.6	11.1	1.6	0.0
Agriculture, forestry, and fishery workers	0.5	10.8	31.1	36.5	17.6	3.4	0.2
Manual workers	0.7	13.5	32.8	36.7	14.3	2.0	0.1
Unemployed persons	1.9	13.6	32.1	36.9	13.7	1.7	0.0
2010							
Professional and engineering workers	0.0	4.4	28.5	41.1	22.2	3.7	0.1
Administrative and managerial workers	0.0	2.4	17.6	35.1	35.5	9.0	0.3
Clerical workers	0.1	4.2	24.5	40.4	26.6	4.1	0.1
Sales workers	0.4	9.2	31.9	35.4	19.5	3.5	0.1
Service workers	0.5	10.7	32.0	35.2	18.3	3.2	0.1
Security workers	0.9	9.8	34.4	35.2	17.8	1.9	0.0
Agriculture, forestry, and fishery workers	0.2	8.5	30.7	36.3	19.6	4.6	0.1
Manual workers	0.7	13.4	29.7	33.3	19.5	3.4	0.0
Unemployed persons	1.7	12.2	29.1	34.4	19.5	3.0	0.1
2015							
Professional and engineering workers	0.0	3.2	25.8	40.6	24.8	5.4	0.2
Administrative and managerial workers	0.0	1.7	14.9	35.1	34.8	12.7	0.8
Clerical workers	0.1	3.4	22.3	39.6	27.6	6.8	0.2
Sales workers	0.3	7.2	28.1	38.6	20.9	4.8	0.1
Service workers	0.6	9.8	28.6	35.9	20.7	4.3	0.1
Security workers	1.0	8.7	33.2	36.7	16.8	3.6	0.0
Agriculture, forestry, and fishery workers	0.1	5.2	25.7	36.7	26.2	6.2	0.1
Manual workers	0.6	11.3	29.4	33.0	20.7	4.9	0.1
Unemployed persons	1.9	11.0	26.3	34.4	21.3	5.0	0.1

**Table 3:** Low birth weight rates by maternal age group for each year in all of Japan.

	Year				
Maternal Age Group	1995	2000	2005	2010	2015
<20 years	9.46	9.56	9.57	10.69	10.00
20–24 years	7.73	8.48	8.67	8.96	8.98
25–29 years	7.18	8.30	8.81	8.88	8.68
30–34 years	7.34	8.52	9.58	9.40	9.13
35–39 years	8.52	9.88	11.16	10.72	10.37
40–44 years	11.46	12.56	13.46	13.11	12.30
>44 years	13.87	14.71	23.41	19.60	17.70

**Table 4:** Standardized low birth weight ratio and its 95% confidence intervals by maternal occupation for each year.

	Year				
Maternal Occupation	1995	2000	2005	2010	2015
Professional and engineering workers	1.011 (0.986, 1.037)	1.039 (1.017, 1.062)	1.030 (1.009, 1.052)	1.018 (0.999, 1.036)	0.990 (0.974, 1.006)
Administrative and managerial workers	0.922 (0.812, 1.042)	0.960 (0.851, 1.080)	1.073 (0.965, 1.189)	0.983 (0.898, 1.074)	0.981 (0.897, 1.070)
Clerical workers	1.052 (1.027, 1.077)	1.043 (1.020, 1.066)	1.046 (1.024, 1.069)	1.031 (1.012, 1.051)	1.016 (0.998, 1.034)
Sales workers	0.959 (0.912, 1.008)	1.016 (0.969, 1.065)	0.966 (0.921, 1.013)	0.988 (0.949, 1.029)	0.989 (0.954, 1.024)
Service workers	1.086 (1.035, 1.140)	1.042 (0.996, 1.088)	1.009 (0.972, 1.047)	1.018 (0.988, 1.048)	1.034 (1.009, 1.060)
Security workers	1.048 (0.860, 1.264)	0.928 (0.778, 1.098)	0.933 (0.806, 1.073)	0.851 (0.737, 0.977)	0.845 (0.739, 0.961)
Agriculture, forestry, and fishery workers	0.892 (0.811, 0.978)	0.872 (0.781, 0.971)	0.881 (0.785, 0.985)	0.924 (0.829, 1.026)	0.865 (0.776, 0.961)
Manual workers	1.057 (1.010, 1.105)	1.071 (1.021, 1.123)	1.123 (1.069, 1.180)	1.096 (1.047, 1.146)	1.072 (1.027, 1.118)
Unemployed persons	1.002 (0.995, 1.010)	0.996 (0.989, 1.003)	0.992 (0.985, 1.000)	0.995 (0.987, 1.003)	0.988 (0.979, 0.996)

## Data Availability

Publicly available datasets were analyzed in this study. This data can be found here: (The Vital Statistics: https://www.e-stat.go.jp/stat-search/files?page=1&toukei=00450011 accessed on 28 June 2021).
